# Case report and a brief review: Analysis and challenges of prenatal imaging phenotypes and genotypes in Joubert syndrome

**DOI:** 10.3389/fgene.2022.1038274

**Published:** 2022-11-18

**Authors:** Ling-Xi Huang, Xian-Gui Lu, Jiao-Xiang Liu, Ling Xu, Ning Shang, Li Guo, Yan-Chun OuYang

**Affiliations:** ^1^ Department of Ultrasound, Guangdong Women and Children Hospital, Guangzhou, China; ^2^ Department of Physical Examination, Guangdong Women and Children Hospital, Guangzhou, China; ^3^ Department of Medical Genetic, Guangdong Women and Children Hospital, Guangzhou, China

**Keywords:** Joubert syndrome, phenotypes, genotypes, prenatal, imaging

## Abstract

Prenatal imaging phenotypes and genotypes were analyzed in 13 cases prenatally diagnosed with Joubert syndrome (JS), all of which underwent magnetic resonance imaging (MRI), ultrasound, and genetic testing. Prenatal MRI diagnosed 10 cases as JS with a typical molar tooth sign (MTS), while prenatal ultrasound diagnosed or suspiciously diagnosed 11 cases as JS with typical or mild MTS in 10 cases. Mutations in JS-related genes and other prenatal JS imaging phenotypes were identified in 10 cases, including OFD1 in two cases [cerebellar vermis (CV) absence, posterior fossa dilation, ventriculomegaly, polydactyly, malformations of cortical development (MCD), and persistent left superior vena cava], TMEM67 in two cases (CV absence, polydactyly, hyperechoic kidneys or polycystic kidneys, posterior fossa dilation, and ventriculomegaly), CC2D2A in two cases (CV absence, polydactyly, MCD, agenesis of the corpus callosum, encephalocele and hydrocephalus, ventriculomegaly, and posterior fossa dilation), RPGRIP1L in one case (CV absence), TCTN3 in one case (CV absence, polydactyly, MCD, and posterior fossa dilation), CEP290 in one case (CV absence and polycystic kidney), and NPHP1 in one case (CV absence). The prenatal diagnosis of JS presents a number of challenges, including the variants of unknown significance, the lack of functional assessment in prenatal imaging, unclear phenotype–genotype relationships in prenatal evaluation, and the incorrect identification of the JS hallmark, the MTS, in prenatal imaging, especially on ultrasound. Although combined MRI, ultrasound, and exome sequencing could help improve the prenatal diagnosis of JS, there still exist significant challenges.

## Introduction

JS is considered an archetypal ciliopathy (Cantagrel et al., 2008). To date, JS is known to be caused by pathogenic variants in 34 genes, 33 autosomal recessive genes and one X-linked gene (Adam et al., 1993). Advancements in molecular diagnostics and imaging technology have facilitated the progress of prenatal diagnosis and genetic evaluation of JS. However, the expanded phenotypes have introduced broader overlaps with other ciliopathies, while the value of prenatal imaging in the diagnosis of JS remains unclear, presenting challenges in the prenatal diagnosis of JS, which we discuss in this study.

## Materials and methods

In the present study, all cases were collected from Guangdong Women and Children Hospital, which is a single-center study. An ultrasound was performed due to a routine pregnancy examination in our center or abnormal ultrasound findings at other centers and referred to our center. In our center, prenatal ultrasound was performed first. When abnormalities (such as cranial abnormalities) were found, genetic consultation was conducted to determine whether prenatal MRI and exome sequencing should be performed. Ultrasonic and MRI diagnostic criteria for JS: MTS are shown in the axial view at the midbrain and pons levels. MTS comprises an abnormally deep interpeduncular fossa; prominent, straight, and thickened superior cerebellar peduncles; and hypoplasia of the vermis, the midline portion of the cerebellum (Maria et al., 1997) (Maria et al., 1999). Inclusion criteria are as follows: 1. all cases performed prenatal MRI, ultrasound, chromosomal microarray, and exome sequencing; 2. the case was diagnosed as JS by prenatal ultrasound or MRI; and 3. an informed consent was signed in all the cases. A total of 13 cases were included in the present study.

## Results

Ultrasound of targeted imaging for fetal anomalies was executed in 133,490 cases between January 2014 and January 2022 in our center. A total of 28 cases of JS were diagnosed by ultrasound and 27 cases by MRI. Exome sequencing identified disease-related gene variants in 10 cases but not in 3 cases. The present study included 13 cases prenatally diagnosed with JS. Thirteen cases underwent prenatal MRI, ultrasound (Table 1), chromosomal microarray, and exome sequencing in that order and were classified according to the American College of Medical Genetics and Genomics (ACMG) (Table 2). In total, 13 cases of JS were identified in 12 families (case 1 and case 2 came from the same family), three of whom had a pregnancy history with JS. All couples were healthy without consanguinity. Twelve families chose to odinopoeia, where this manipulation was legal in our country (autopsies were performed in three cases), and in one case, the infant was born (case 13).

**TABLE 1:** Prenatal imaging phenotypes and diagnosis of 13 cases’ fetuses.

Case	GA at US diagnosis (weeks)	**Diagnosis (MRI)**	**MRI phenotype**	**Diagnosis (US)**	**US phenotype**	Autopsy
1	25	J	T-MTS, CV absence, PFD, s-ventriculomegaly, and MCD	J	T-MTS, CV absence, PFD, s-ventriculomegaly, polydactyly, and PLSVC	CV absence, polydactyly, and PLSVC
2	30	J	T-MTS, CV absence, and PFD	J	T-MTS, CV absence, PFD, polydactyly, and PLSVC	-
3	17	J	T-MTS and CV absence	J	T-MTS, CV absence, polydactyly, and hyperechoic kidneys	Most of CV absence and polydactyly
4	28	V-D	M-MTS and most of CV absence	S-J	M-MTS and CV absence	-
5	25	J	T-MTS, CV absence, and MCD	J	T-MTS, CV absence, encephalocele, and hydrocephalus	-
6	24	J	T-MTS, CV absence, PFD, and MCD	J	T-MTS, CV absence, PFD, MCD, and polydactyly	-
7	18	J	T-MTS and CV absence	J	T-MTS, CV absence, and polycystic kidneys	-
8	25	J	T-MTS, CV absence, PFD, s-ventriculomegaly, and polycystic kidneys	DW	T-MTS, most of CV absence, and polycystic kidneys	CV absence, polycystic kidney, and VSD
9	23	DW/J	T-MTS, CV absence, MCD, and agenesis of the corpus callosum	S-J	M-MTS, CV absence, PFD, s-ventriculomegaly, MCD, and polydactyly	CV absence, MCD, polydactyly, agenesis of the corpus callosum, and s-ventriculomegaly
10	24	V-D	Most of CV absence	S-J	Most of CV absence	-
11	27	J	T-MTS, CV absence, and MCD	BP	polydactyly	-
12	23	J	T-MTS and most of CV absence	S-J	M-MTS and most of CV absence	-
13	24	N	s-Ventriculomegaly and ependymal cyst	S-J	M-MTS, most of CV absence, and s-ventriculomegaly	-

GA, gestational age; US, ultrasound; CV, cerebellar vermis; PFD, posterior fossa dilation; MCD, malformation of cortical development; PLSVC, persistent left superior vena cava; J, diagnosed with JS; S-J, JS suspected; BP, Blake’s pouch cyst; DW, Dandy–Walker syndrome; V-D, vermis dysplasia; s-ventriculomegaly, slight ventriculomegaly; T-MTS, typical MTS; M-MTS, mild MTS; N, normal.

**TABLE 2:** Results of 13 cases in WES.

Case	Gene	Mutation	**Homozygous/heterozygous**	ACMG classification	OMIM ID
1	F: Normal				
	M: OFD1	c.2848A>T (p.K950*)	Heterozygous	LPV	300804
	Fe: OFD1	c.2848A>T (p.K950*)	Homozygous	LPV	
2	F: Normal				
	M: OFD1	c.2848A>T (p.K950*)	Heterozygous	LPV	300804
	Fe: OFD1	c.2848A>T (p.K950*)	Homozygous	LPV	
3	F: TMEM 67	c.224G>A (p.Gly75GLu)	Heterozygous	VUS	610688
	M: TMEM 67	c.2345A>G (p.His782Arg)	Heterozygous	VUS	
	Fe: TMEM 67	c.224G>A (p.Gly75GLu) and c.2345A>G (p.His782Arg)	Heterozygous	VUS	
4	F: RPGRIP1L	c.1641dupA (p. Val548SerfsTer9)	Heterozygous	VUS	611560
	M: RPGRIP1L	c.3764T>C (p. Ile1255Thr)	Heterozygous	VUS	
	Fe: RPGRIP1L	c.1641dupA (p. Val548SerfsTer9) and c.3764T>C (p. Ile1255Thr)	Heterozygous	VUS	
5	F: CC2D2A	c.3688C>T (p.R1230*)	Heterozygous	LPV	216360/6,122
	M: CC2D2A	c.418insGAGGGAGGAGCCAAGA?	Heterozygous	LPV	85/612284
	Fe: CC2D2A	c.3688C>T (p.R1230*) and c.418insGAGGGAGGAGCCAAGA_?	Heterozygous	LPV	
6	F: TCTN3	c.1441dupT (p.C481Lfs*133)	Heterozygous	LPV	614518
	M: TCTN3	c.1441dupT (p.C481Lfs*133)	Heterozygous	LPV	
	Fe: TCTN3	c.1441dupT (p.C481Lfs*133)	Homozygous	LPV	
7	F: CEP290	c.4303–2A>T	Heterozygous	VUS	610188
	M: CEP290	c.2569A>T (K857X)	Heterozygous	VUS	
	Fe: CEP290	c.4303–2A>T and c.2569A>T (K857X)	Heterozygous	VUS	
8	F: TMEM 67	c.1175C>G	Heterozygous	LPV	610688
	M: TMEM 67	c.1250A>G	Heterozygous	LPV	
	Fe: TMEM 67	c.1175C>G and c.1250A>G	Heterozygous	LPV	
9	F: CC2D2A	Loss of heterozygosity in exon 20–21	Heterozygous	LPV	216360/6,122
	M: CC2D2A	c.2003 + 2T>C	Heterozygous	LPV	85/612284
	Fe: CC2D2A	Loss of heterozygosity in exon 20–21 and c.2003 + 2T>C	Heterozygous	LPV	
10	F: NPHP1	2q13 (chr2:110873257–110962571)	Heterozygous	PV	609583/2,561
	M: NPHP1	2q13 (chr2:110873257–110962571)	Heterozygous	PV	00/266900
	Fe: NPHP1	2q13 (chr2:110873257–110962571)	Homozygous	PV	
11	Fe: Negative				
12	Fe: Negative				
**13**	Fe: Negative				

F, father; M, mother; Fe, fetus; LPV, likely pathogenic variant; VUS, variant of unknown significance; PV, pathogenic variant; J, Joubert syndrome; Negative, no JS-related pathogenic genes.

No anomalies were found in the 13 cases by chromosomal microarray. Exome sequencing identified disease-related gene variants in 10 cases. Of the variants in these 10 cases, one was classified as pathogenic, six as likely pathogenic, and three as variants of unknown significance according to ACMG guidelines. Seven cases involved autosomal recessive variants, and two involved hemizygous OFD1 loss-of-function mutations.

The prenatal diagnosis by ultrasound and MRI was consistent in eight cases (diagnosis of JS, 8/13) and inconsistent in five cases (5/13). Prenatal MRI of the 13 cases diagnosed 10 as JS with typical MTS (one case was diagnosed as Dandy–Walker syndrome without MTS at 23 weeks, while JS with typical MTS was diagnosed at 28 weeks) and 2 as CV dysplasia (retrospective analysis showed mild MTS in one case and no MTS in the other); no abnormalities were found in one case (case 13). Prenatal ultrasound of the 13 cases diagnosed or suspiciously diagnosed 11 as JS with 10 typical or mild MTS, 1 as Dandy-Walker syndrome (typical MTS shown in retrospective analysis), and 1 as Blake’s pouch cyst without MTS.

In total, two cases (case 1 and 2) of OFD1 mutations were diagnosed as JS by prenatal MRI and ultrasound with typical MTS, CV absence, posterior fossa dilation, ventriculomegaly, polydactyly, MCD, and persistent left superior vena cava. One case (case 3) of TMEM67 mutations was diagnosed as JS by prenatal MRI and ultrasound with typical MTS, CV absence, hyperechoic kidneys, and polydactyly. One case (case 8) of TMEM67 mutations was diagnosed as JS by prenatal MRI with typical MTS, CV absence, posterior fossa dilation, ventriculomegaly, and polycystic kidneys, and as Dandy–Walker syndrome by prenatal ultrasound with typical MTS (retrospective analysis), most of CV absence, and polycystic kidneys. One case (case 4) of RPGRIP1L mutations was diagnosed as CV dysplasia by prenatal MRI (mild MTS found in retrospective analysis) and suspiciously diagnosed as JS with mild MTS and CV absence by ultrasound. One case (case 5) of CC2D2A mutations was diagnosed as JS by prenatal MRI and ultrasound with typical MTS, CV absence, MCD, encephalocele, and hydrocephalus. One case (case 9) of CC2D2A mutations was suspiciously diagnosed as JS by prenatal ultrasound with mild MTS, CV absence, posterior fossa dilation, ventriculomegaly, MCD, and polydactyly and diagnosed as Dandy–Walker without MTS by prenatal MRI at 23 weeks but as JS with typical MTS, CV absence, MCD, and agenesis of the corpus callosum at 28 weeks. One case (case 6) of TCTN3 mutations was diagnosed as JS by prenatal MRI and ultrasound with typical MTS, CV absence, polydactyly, MCD, and posterior fossa dilation. One case (case 7) of CEP290 mutations was diagnosed as JS by prenatal MRI and ultrasound with typical MTS, CV absence, and polycystic kidneys. One case (case 10) of NPHP1 mutations was diagnosed as CV dysplasia by prenatal MRI and suspiciously diagnosed as JS with most of CV absence by prenatal ultrasound.

## Discussion

JS is a complex malformation of the midbrain and hindbrain that results in the MTS. MTS is considered the hallmark and distinctive imaging feature of JS (Maria et al., 1997) (Maria et al., 1999). The diagnosis of JS is based on three primary criteria: MTS, hypotonia in infancy with later development of ataxia, and developmental delay or intellectual disability (Adam et al., 1993–2022). Although the diagnostic criteria for JS continued to evolve, most authors concur that the neuroradiological finding of the MTS is obligatory (Adam et al., 1993). In the past, the prevalence between 1/80,000 and 1/100,000 live births was reported by many authors (Juric-Sekhar et al., 2012). However, it is likely underestimated because of legally induced labors in some countries and the limited awareness of the MTS (Juric-Sekhar et al., 2012). The present study revealed a high uniformity between prenatal imaging phenotypes and genetic sequencing in nine cases, whose prevalence was approximately 1/1,4832. The prevalence of the present study was much higher than reported in previous studies because our center was a provincial referral center for abnormal fetuses. In addition, the live births and induced labor were included in the present study.

**FIGURE 1:**
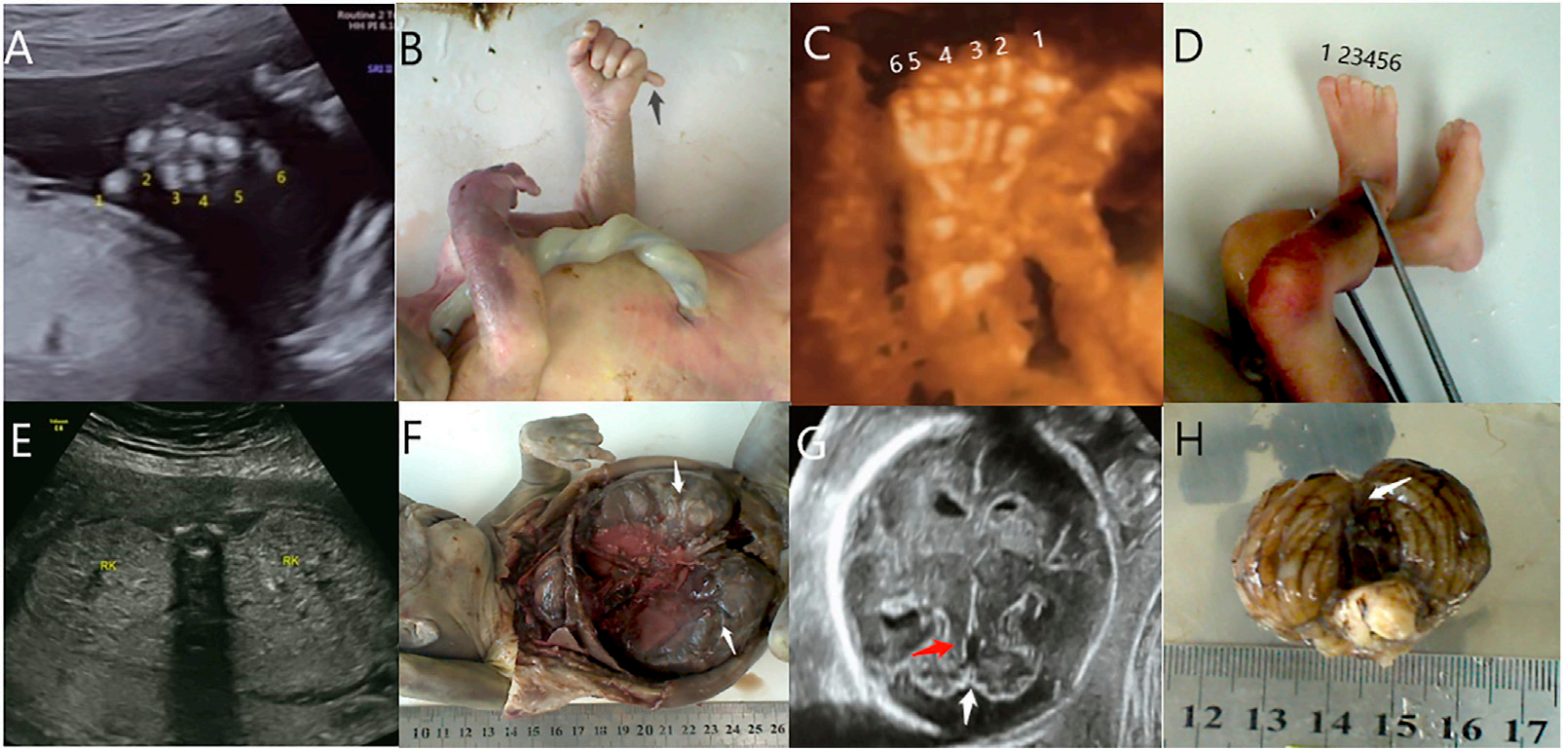
Polydactyly (black arrow) was detected by ultrasound **(A)** and autopsy **(B)** in case 1. Polydactyly was detected by ultrasound **(C)** and autopsy **(D)** in case 3. Polycystic kidneys (white arrow) were detected by ultrasound **(E)** and autopsy **(F)** in case 8. **(G)** The mild MTS (red arrow) and CV absence (white arrow) were detected by ultrasound in case 9. **(H)** CV absence (white arrow) was detected by an autopsy in case 9.

The OFD1 gene that may play a role in cortical and heart development (Zhang et al., 2020) is involved in JS. OFD1 mutations are inherited in an X-linked recessive fashion; males are affected, while female carriers are asymptomatic (Pezzella et al., 2022). Encephalocele, hydrocephalus, macrocephaly, MCD, polydactyly, renal cystic disease, retinal disease, and tetralogy of Fallot are observed in OFD1-related JS (Coene et al., 2009) (Field et al., 2012) (Zhang et al., 2020). We identified a hemizygous OFD1 variant c.2848A>T (p.K950*), classified as likely pathogenic, in two male fetuses (cases 1 and 2), where the mother was the carrier of the mutation. These two cases were diagnosed as JS by prenatal MRI and ultrasound, based on typical MTS, CV absence, posterior fossa dilation, ventriculomegaly, polydactyly (Figures 1A,B), MCD, and persistent left superior vena cava. These prenatal imaging features were consistent with JS phenotypes. This variant might be the potential genetic etiology of the JS.

TMEM67 mutations are most prevalent in the Japanese (25.9%), North American (22.2%), and North European (23.8%) populations (Suzuki et al., 2016) (Brooks et al., 2018). Hepatic disease, renal disease, ocular coloboma, and polydactyly are phenotypes observed in JS associated with TMEM67 mutations (Iannicelli et al., 2010) (Doherty et al., 2010). To date, liver involvement, including liver fibrosis (main feature), hepatomegaly, and the elevation of liver enzymes, is found in about 70%–80% of TMEM67-related JS, representing the strongest gene–phenotype association among all JS types (Iannicelli et al., 2010) (Tsurusaki et al., 2015) (Romani et al., 2013). However, these hepatic features are harder to observe prenatally than craniocerebral, renal, and polydactyly abnormalities. Here, we identified two cases with TMEM67 variants. Case 3 carried heterozygous variants of unknown significance, with a paternally inherited c.224G>A (p.Gly75GLu) variant and a maternally inherited c.2345A>G (p.His782Arg) variant. This case was diagnosed as JS by prenatal MRI and ultrasound based on typical MTS, CV absence, hyperechoic kidney, and polydactyly (Figures 1C,D), consistent with JS phenotypes. Combined with the genetic results, a diagnosis of JS was inclined. Case 8 carried heterozygous likely pathogenic variants, with a paternally inherited c.1175C>G variant and a maternally inherited c.1250A>G variant. This case was diagnosed as JS by prenatal MRI based on typical MTS, CV absence, posterior fossa dilation, ventriculomegaly, and polycystic kidneys and as Dandy–Walker syndrome by prenatal ultrasound with typical MTS (retrospective analysis), most of CV absence, and polycystic kidneys (Figures 1E,F). These prenatal imaging features were consistent with JS phenotypes. The variants of case 3 and case 8 might be the potential genetic etiologies of the JS.

The absence of the C2 domain may lead to the abnormal development of the nervous system in individuals with CC2D2A mutations, such as encephalocele, ventriculomegaly, seizures, and mental retardation (Bachmann-Gagescu et al., 2012) (Basel-Vanagaite et al., 2006). Retinal, renal, oculorenal, hepatic, and central nervous system (CNS) abnormalities are observed in JS associated with CC2D2A mutations (Adam et al., 1993) (Gorden et al., 2008). We identified two cases with CC2D2A variants, which were classified as likely pathogenic. Case 5 was heterozygous with a paternally inherited c.3688C>T (p.R1230*) variant and a maternally inherited c.418insGAGGGAGGAGCCAAGA_? variant. This case was diagnosed as JS by prenatal MRI and ultrasound based on typical MTS, CV absence, MCD, encephalocele, and hydrocephalus. These prenatal imaging features were consistent with JS phenotypes. This variant might be the potential genetic etiology of the JS. Case 9 was heterozygous with a paternally inherited loss of heterozygosity in exons 20 and 21 of the *CC2D2A* gene and a maternally inherited c.2003 + 2T>C variant. This case was suspiciously diagnosed as JS by prenatal ultrasound based on mild MTS (Figure 1G), CV absence (Figures 1G,H), posterior fossa dilation, ventriculomegaly, MCD, and polydactyly. The case was diagnosed as Dandy–Walker syndrome without MTS by prenatal MRI at 23 weeks and as JS with typical MTS, CV absence, MCD, and agenesis of the corpus callosum at 28 weeks. Combined with the prenatal imaging features, this variant cannot be excluded as a potential genetic etiology of JS.

NPHP1 mutations can cause juvenile NPHP type 1, Cogan syndrome, and JS, with retinal, renal, and oculorenal involvement (Adam et al., 1993). NPHP1 mutations give rise to a milder form of JS as they produce mild MTS (elongated but thin superior cerebellar peduncles and milder vermis hypoplasia) without severe clinical symptoms (Parisi et al., 2004). In the Parisi study, mild MTS was visible in the subjects of NPHP1 deletion. Moreover, they found that MTS was visible on the MRI of the older child, but this scan was interpreted initially as normal (Parisi et al., 2004). Also, they established, retrospectively, the presence of MTS nonspecific delays prior to the onset of NPHP (Parisi et al., 2004). Hence, the case of NPHP1 mutation was difficult to diagnose with certainty without MTS, especially at the prenatal stage, probably because NPHP1 is only indirectly involved in primary cilium formation and function. Case 10 was homozygous for the loss of [2q13 (chr2:110873257–110962571)] at NPHP1, classified as pathogenic. This case was diagnosed as CV dysplasia by prenatal MRI and suspiciously diagnosed as JS with most of CV absence by prenatal ultrasound without other imaging phenotypes. However, despite the absence of MTS and other phenotypes associated with ciliopathies, based on the phenotypic characteristics of the NPHP1 variation, case 10 could not completely rule out JS.

Mutations in RPGRIP1L may cause a wide range of symptoms that involve many organs, including retinal, renal, hepatic, and oculorenal symptoms, polydactyly, and encephalocele (Adam et al., 1993). Case 4 was heterozygous for RPGRIP1L variants, classified as variants of unknown significance, with a paternally inherited c.1641dupA (p.Val548SerfsTer9) variant and a maternally inherited c.3764T>C (p.Ile1255Thr) variant. This case was diagnosed as CV dysplasia by prenatal MRI (mild MTS found in retrospective analysis) and suspiciously diagnosed as JS with mild MTS and CV absence by ultrasound without other imaging phenotypes. The genetic results and atypical prenatal imaging features weakly supported a diagnosis of JS.

TCTN3 mutations, which are less common molecular causes of JS, give rise to scoliosis, polydactyly, oral findings, horseshoe kidneys, and VSD (Thomas et al., 2012). Case 6 was homozygous for the [c.1441dupT (p.C481Lfs*133)] variant at TCTN3, which was classified as likely pathogenic. This case was diagnosed as JS by prenatal MRI and ultrasound, based on typical MTS, CV absence, polydactyly, MCD, and posterior fossa dilation. This case had the same mutation as the latter fetus and pregnancy history of CV dysplasia. The prenatal imaging features were consistent with JS phenotypes. The variant might be the potential genetic etiology of the JS.

CEP290 is termed “a gene with many faces” for its broad phenotypic spectrum, which involves retinal dystrophy, renal disease, hepatic disease, cardiac disease, encephalocele, and situs inversus (Adam et al., 1993; Valente et al., 2008; Coppieters et al., 2010). Case 7 was heterozygous for CEP290 variants, classified as variants of unknown significance, with a paternally inherited c.4303–2A>T variant and a maternally inherited c.2569A>T (K857X) variant. This case was diagnosed as JS by prenatal MRI and ultrasound, based on typical MTS, CV absence, and polycystic kidneys. The prenatal imaging features were consistent with JS phenotypes. The variant might be the potential genetic etiology of the JS.

Remarkably, no mutations were found in three cases in JS-related genes by exome sequencing. In case 13, ventriculomegaly and ependymal cyst were found on prenatal MRI, while prenatal ultrasound suspiciously diagnosed the case as JS with mild MTS, most of CV absence, and ventriculomegaly. At 1 year after birth, no abnormalities were found on MRI, and there were no clinical symptoms at present. Therefore, JS diagnosis was not supported for case 13. The other two cases (cases 11 and 12) were diagnosed as JS by prenatal MRI based on typical MTS and CV absence; case 11 presented polydactyly and MCD, while case 12 did not exhibit other imaging phenotypes. Therefore, the diagnosis was uncertain for these two cases, and further studies are needed in both families.

**FIGURE 2:**
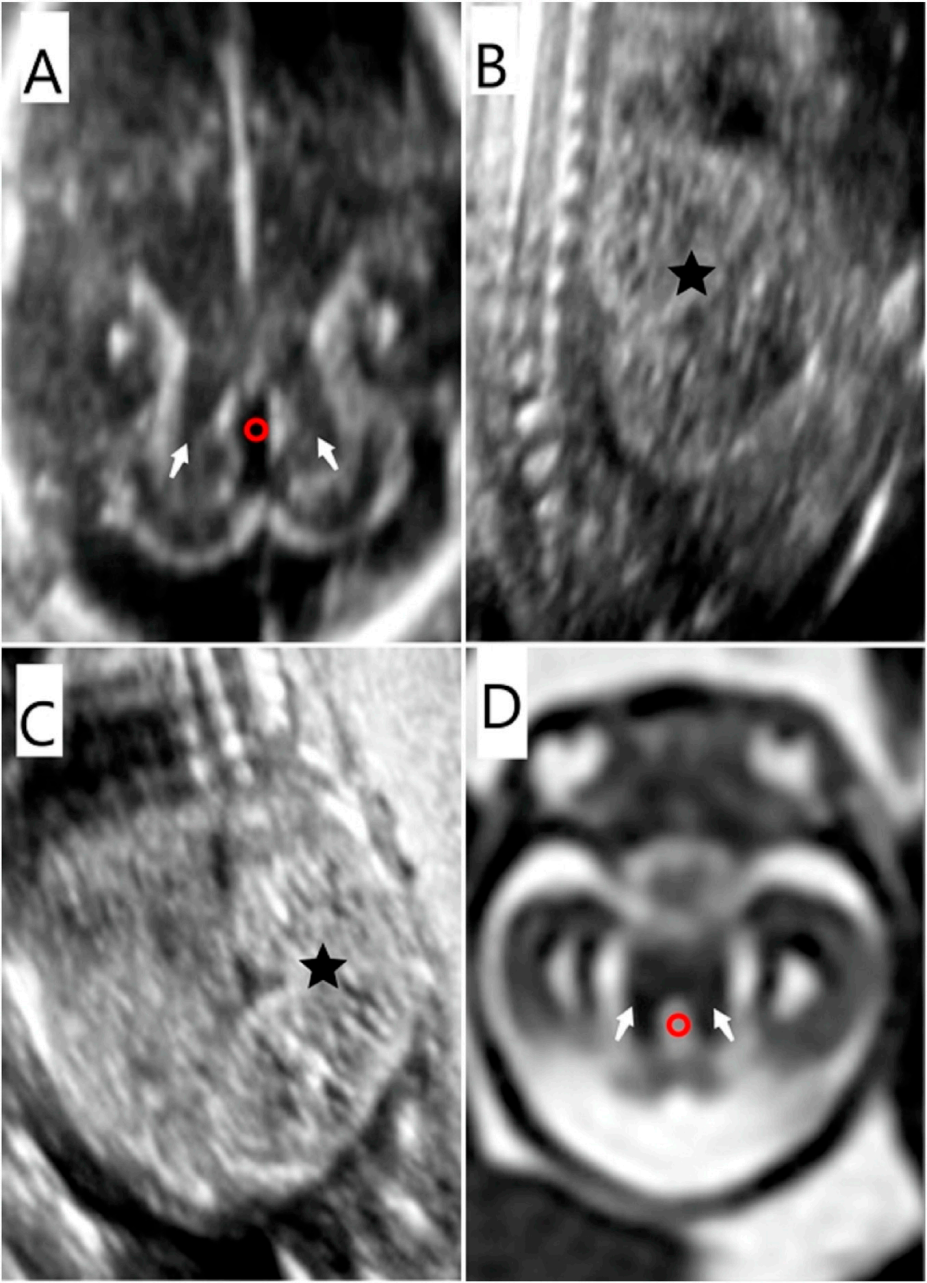
Prenatal imaging features of the fetus in case 7. **(A) (D)** MTS (white arrow) was detected by US and MRI. The fourth ventricle presented as “elongated” (red circle) on US and MRI. **(B) (C)** Bilateral polycystic kidneys (★) were identified by ultrasound.

There are several challenges concerning the prenatal diagnosis of JS. They are as follows:

(1) In a prospective cohort study of 610 fetuses with a broad range of structural anomalies, diagnostic or potentially clinically relevant variants were identified in 76 (12.5%) fetuses (Lord et al., 2019). Supplementing chromosomal microarray with exome sequencing substantially increased the number of fetuses diagnosed with genetic variants associated with developmental disorder genes (Lord et al., 2019). However, such likely pathogenic variants pose major challenges for prenatal counseling and decision-making. Prenatal exome sequencing testing is a phenotype-driven diagnosis, and fetal phenotype identification depends on ultrasound and MRI. Prenatal imaging should seek as many phenotypes associated with the variants or poor prognoses (such as CV dysplasia) as possible, especially for variants of unknown significance. The more sufficient the phenotypic evidence, the more accurate the prenatal guidance should be. However, ultrasound is not really reliable enough, and an MRI is preferable if available, especially in the aspect of a fetal nervous system.
(2) The diagnosis of JS is primarily based on clinical symptoms, such as hypotonia in infancy with later development of ataxia and developmental delays or intellectual disability. However, such symptoms cannot be assessed or predicted with prenatal imaging, which is a structural rather than a functional examination. Complex phenotypes emerge as infants’ age, and thus, the evaluation of the phenotype–genotype relationship is incomplete in the prenatal period. Different gene variants of JS can lead to varied and complex clinical phenotypes that may not all be present at the time of diagnosis. Some phenotypes present more often in infancy, or even in childhood or later, such as progressive liver fibrosis and nephronophthisis with onset in late childhood or later (Doherty et al., 2010) (Kumada et al., 2004). Therefore, phenotype–genotype relationships cannot be fully identified in prenatal evaluations.
(3) JS shares the same pathogenic genes with other diseases. Meckel syndrome shares at least 18 causative pathogenic genes with JS, which might explain the striking clinical overlap between these two disorders (Adam et al., 1993) (Bachmann-Gagescu et al., 2015). Because of this, it is difficult to distinguish Meckel syndrome from JSRD based on clinical phenotypes and genetic testing, especially prenatally. MTS is an essential sign in distinguishing JS from other disorders. However, not all MTS can be correctly identified by prenatal imaging, especially by ultrasound. First, MTS may be visualized as early as 22 weeks by 2D and 3D ultrasound in fetuses without a prior family history of JS (Quarello et al., 2014); however, the CV may not cover the fourth ventricle before 18 weeks yet, so it remains difficult to detect MTS in an early pregnancy when there is no pregnancy history of JS or phenotypes of ciliopathy (Bromley et al., 1994). In this study, MTS was detected at 17–18 gestational weeks by ultrasound in two cases (case 3 and case 7) (Figures 2A,D). In case 3, the mother had a pregnancy history of JS and polydactyly, and case 7 presented with polycystic kidney disease (Figures 2B,C). Second, the direction of the superior peduncles may vary with the severity of CV hypoplasia and MTS (Poretti et al., 2011), which may be related to different pathogenic genes. Third, the limitations to imaging technology and lack of experience of radiologists may lead to the misdiagnosis of MTS in prenatal imaging. At present, the sensitivity and specificity of prenatal imaging in the diagnosis of JS are unclear.

## Conclusion

To a certain degree, prenatal imaging can identify MTS and identify imaging phenotypes. Despite challenges in the accurate prenatal diagnosis of JS, the development of molecular diagnosis and imaging technology may facilitate the diagnosis of genetic disorders in the future so that better guidance can be provided in prenatal counseling.

## Data Availability

The original contributions presented in the study are included in the article/Supplementary Materials; further inquiries can be directed to the corresponding author.

## References

[B1] Adam M. P. Everman D. B. Mirzaa G. M. Pagon R. A. Parisi M. Glass L. (1993). Gene reviews. Seattle Washington: University of Washington.

[B2] Bachmann-Gagescu R. Dempsey J. C. Phelps I. G. O'Roak B. J. Knutzen D. M. Rue T. C. (2015). Joubert syndrome: A model for untangling recessive disorders with extreme genetic heterogeneity. J. Med. Genet. 52 (8), 514–522. 10.1136/jmedgenet-2015-103087 26092869 PMC5082428

[B3] Bachmann-Gagescu R. Ishak G. E. Dempsey J. C. Adkins J. O'Day D. Phelps I. G. (2012). Genotype-phenotype correlation in CC2D2A-related Joubert syndrome reveals an association with ventriculomegaly and seizures. J. Med. Genet. 49 (2), 126–137. 10.1136/jmedgenet-2011-100552 22241855

[B4] Basel-Vanagaite L. Attia R. Yahav M. Ferland R. J. Anteki L. Walsh C. A. (2006). The CC2D1A, a member of a new gene family with C2 domains, is involved in autosomal recessive nonsyndromic mental retardation. J. Med. Genet. 43 (3), 203–210. 10.1136/jmg.2005.035709 16033914 PMC2563235

[B5] Bromley B. Nadel A. S. Pauker S. Estroff J. A. Benacerraf B. R. (1994). Closure of the cerebellar vermis: Evaluation with second trimester US. Radiology 193 (3), 761–763. 10.1148/radiology.193.3.7972820 7972820

[B6] Brooks B. P. Zein W. M. Thompson A. H. Mokhtarzadeh M. Doherty D. A. Parisi M. (2018). Joubert Syndrome: Ophthalmological findings in correlation with genotype and hepatorenal disease in 99 patients prospectively evaluated at a single center. Ophthalmology 125 (12), 1937–1952. 10.1016/j.ophtha.2018.05.026 30055837 PMC8932443

[B7] Cantagrel V. Silhavy J. L. Bielas S. L. Swistun D. Marsh S. E. Bertrand J. Y. (2008). Mutations in the cilia gene ARL13B lead to the classical form of Joubert syndrome. Am. J. Hum. Genet. 83 (2), 170–179. 10.1016/j.ajhg.2008.06.023 18674751 PMC2495072

[B8] Coene K. L. Roepman R. Doherty D. Afroze B. Kroes H. Y. Letteboer S. J. (2009). OFD 1 is mutated in X-linked Joubert syndrome and interacts with LCA5-encoded lebercilin. Am. J. Hum. Genet. 85 (4), 465–481. 10.1016/j.ajhg.2009.09.002 19800048 PMC2756557

[B9] Coppieters F. Lefever S. Leroy B. P. Baere E. D. (2010). CEP290, a gene with many faces: Mutation overview and presentation of CEP290base. Hum. Mutat. 31 (10), 1097–1108. 10.1002/humu.21337 20690115

[B10] Doherty D. Parisi M. A. Finn L. S. Gunay-Aygun M. Al-Mateen M. Bates D. (2010). Mutations in 3 genes (MKS3, CC2D2A and RPGRIP1L) cause COACH syndrome (Joubert syndrome with congenital hepatic fibrosis). J. Med. Genet. 47 (1), 8–21. 10.1136/jmg.2009.067249 19574260 PMC3501959

[B11] Field M. Scheffer I. E. Gill D. Wilson M. Christie L. Shaw M. (2012). Expanding the molecular basis and phenotypic spectrum of X-linked Joubert syndrome associated with OFD1 mutations. Eur. J. Hum. Genet. 20 (7), 806–809. 10.1038/ejhg.2012.9 22353940 PMC3376274

[B12] Gorden N. T. Arts H. H. Parisi M. A. Coene K. L. Letteboer S. J. van Beersum S. E. (2008). CC2D2A is mutated in Joubert syndrome and interacts with the ciliopathy-associated basal body protein CEP290. Am. J. Hum. Genet. 83 (5), 559–571. 10.1016/j.ajhg.2008.10.002 18950740 PMC2668034

[B13] Iannicelli M. Brancati F. Mougou-Zerelli S. Mazzotta A. Thomas S. Elkhartoufi N. (2010). Novel TMEM67 mutations and genotype-phenotype correlates in meckelin-related ciliopathies. Hum. Mutat. 31 (5), E1319–E1331. 10.1002/humu.21239 20232449 PMC2918781

[B14] Juric-Sekhar G. Adkins J. Doherty D. Hevner R. F. (2012). Joubert syndrome: Brain and spinal cord malformations in genotyped cases and implications for neurodevelopmental functions of primary cilia. Acta Neuropathol. 123 (5), 695–709. 10.1007/s00401-012-0951-2 22331178

[B15] Kumada S. Hayashi M. Arima K. Nakayama H. Sugai k. Sasaki M. (2004). Renal disease in Arima syndrome is nephronophthisis as in other Joubert-related Cerebello-oculo-renal syndromes. Am. J. Med. Genet. A 131 (1), 71–76. 10.1002/ajmg.a.30294 15384098

[B16] Lord J. McMullan D. J. Eberhardt R. Y. Rinck G. Hamilton S. J. Quinlan-Jones E. (2019). Prenatal exome sequencing analysis in fetal structural anomalies detected by ultrasonography (PAGE): A cohort study. Lancet 393 (10173), 747–757. 10.1016/S0140-6736(18)31940-8 30712880 PMC6386638

[B17] Maria B. L. Hoang K. B. Tusa R. J. Mancuso A. A. Hamed L. M. Quisling R. G. (1997). Joubert syndrome” revisited: Key ocular motor signs with magnetic resonance imaging correlation. J. Child. Neurol. 12 (7), 423–430. 10.1177/088307389701200703 9373798

[B18] Maria B. L. Quisling R. G. Rosainz L. C. Yachnis A. T. Gitten J. C. Dede D. E. (1999). Molar tooth sign in Joubert syndrome: Clinical, radiologic, and pathologic significance. J. Child. Neurol. 14 (6), 368–376. 10.1177/088307389901400605 10385844

[B19] Parisi M. A. Bennett C. L. Eckert M. L. Dobyns W. B. Gleeson J. G. Shaw D. W. (2004). The NPHP1 gene deletion associated with juvenile nephronophthisis is present in a subset of individuals with Joubert syndrome. Am. J. Hum. Genet. 75 (1), 82–91. 10.1086/421846 15138899 PMC1182011

[B20] Pezzella N. Bove G. Tammaro R. Franco B. (2022). Ofd 1: One gene, several disorders. Am. J. Med. Genet. C Semin. Med. Genet. 190 (1), 57–71. 10.1002/ajmg.c.31962 35112477 PMC9303915

[B21] Poretti A. Huisman T. Scheer I. Boltshauser E. (2011). Joubert syndrome and related disorders: Spectrum of neuroimaging findings in 75 patients. AJNR. Am. J. Neuroradiol. 32 (8), 1459–1463. 10.3174/ajnr.A2517 21680654 PMC7964342

[B22] Quarello E. Molho M. Garel C. Couture A. Legac M. P. Moutard M. L. (2014). Prenatal abnormal features of the fourth ventricle in Joubert syndrome and related disorders. Ultrasound Obstet. Gynecol. 43 (2), 227–232. 10.1002/uog.12567 23868831

[B23] Romani M. Micalizzi A. Valente E. M. (2013). Joubert syndrome: Congenital cerebellar ataxia with the molar tooth. Lancet. Neurol. 12 (9), 894–905. 10.1016/S1474-4422(13)70136-4 23870701 PMC3809058

[B24] Suzuki T. Miyake N. Tsurusaki Y. Okamoto N. Alkindy A. Inaba A. (2016). Molecular genetic analysis of 30 families with Joubert syndrome. Clin. Genet. 90 (6), 526–535. 10.1111/cge.12836 27434533

[B25] Thomas S. Legendre M. Saunier S. Bessières B. Alby C. Bonnière M. (2012). TCTN3 mutations cause Mohr-Majewski syndrome. Am. J. Hum. Genet. 91 (2), 372–378. 10.1016/j.ajhg.2012.06.017 22883145 PMC3415538

[B26] Tsurusaki Y. Tanaka R. Shimada S. Shimojima K. Shiina M. Nakashima M. (2015). Novel compound heterozygous LIAS mutations cause glycine encephalopathy. J. Hum. Genet. 60 (10), 631–635. 10.1038/jhg.2015.72 26108146

[B27] Valente E. M. Brancati F. Dallapiccola B. (2008). Genotypes and phenotypes of Joubert syndrome and related disorders. Eur. J. Med. Genet. 51 (1), 1–23. 10.1016/j.ejmg.2007.11.003 18164675

[B28] Zhang Y. W. Qu H. B. Long N. Leng X. Y. Liu Y. Q. Yang Y. (2020). A rare mutant of OFD1 gene responsible for Joubert syndrome with significant phenotype variation. Mol. Genet. Genomics. 296 (1), 33–40. 10.1007/s00438-020-01726-1 32944789

